# Mining genetic loci and candidate genes related to salt tolerance traits in soybean

**DOI:** 10.1038/s41598-025-08702-y

**Published:** 2025-07-23

**Authors:** Rui Tian, Dai Han, Xiaolei Shi, Qimike Shan, Sunlei Ding, Yucheng Wu, Jinbo Zhang, Yongliang Yan

**Affiliations:** 1 Crop Institute, Xinjiang Academy of Agricultural Sciences, Nanchang Road 403, Urumqi City, 830091 Xinjiang Uygur Autonomous Regions People’s Republic of China; 2 National Central Asian Characteristic Crop Germplasm Resources Medium-term Gene Bank (Urumqi), Urumqi City, 830091 Xinjiang Uygur Autonomous Regions People’s Republic of China; 3 College of Agriculture, Xinjiang Agricultural University, Urumqi City, 830052 Xinjiang Uygur Autonomous Regions People’s Republic of China; 4 Xinjiang Institute of Ecology and Geography, Chinese Academy of Sciences, Urumqi City, 830011 People’s Republic of China; 5 Key Laboratory of Ecological Safety and Sustainable Development in Arid Lands, Chinese Academy of Sciences, Urumqi City, 830011 People’s Republic of China

**Keywords:** Soybean, Salt tolerance-related traits, GWAS, Candidate genes, Genetics, Agricultural genetics

## Abstract

Soybean is an important crop worldwide that provides ~ 50% oil for humans. Salinity is a major abiotic stress that inhibits soybean growth and yield. Dissecting the genetic basis of salt tolerance is an effective way for soybean plants to combat salt-related yield losses. In this study, the variety salt tolerance index (STIv) of a natural population of 140 soybean germplasms was calculated in terms of plant height (PH), leaf area (LA), shoot fresh weight (SFW) and shoot dry weight (SDW), which were measured under normal condition and in a 1.50% NaCl solution. GWAS analysis was subsequently conducted on the basis of STIv and 150 K SNP markers of “Zhongdouxin-1”. The results revealed that 365 significant SNPs located on 19 chromosomes (excluding Gm03) were associated with STIv. Among them, 108 SNPs were associated with LA-STIv, 71 SNPs associated with PH-STIv, 95 SNPs associated with SDW-STIv and 91 SNPs associated with SFW-STIv. A total of 333 genes were identified according to the flanking region (150 kb) of the significant SNPs. 333 genes were identified. Based on gene functional annotations, SNP mutations, and RNA expressions, nine causal genes responsible for soybean salt tolerance were identified. Thus, the significantly associated SNPs and candidate genes detected in this study might provide novel insights into soybean salt tolerance in breeding programs.

**Supplementary Information:**

The online version contains supplementary material available at 10.1038/s41598-025-08702-y.

## Introduction

Soybean is an important oil crop worldwide that plays a vital role in the global economy. Salinity is a major abiotic stress that inhibits seed germination, seedling establishment^1,2^, and even yield for many crops^3–6^. According to the Food and Agriculture Organization (FAO), salinity affects 60 million hectares of irrigated arable land worldwide. Soil salinity is a growing problem that renders 0.3–1.5 million hectares of farmland out of production and reduces the production potential of an additional 20–46 million hectares annually^7–9^. With the increase in the degree of salinization of arable land, improving soybean salt tolerance is becoming an important means for combating salt-related yield losses.

Compared with other crops, soybean is identified as a salt-sensitive species^10^. Harboring salt tolerance—related genes was the most effective strategies for enhancing soybean and crop productivity under saline conditions^11^. To clarify the inheritance of salt tolerance in soybean, many scientists have implemented linkage analysis to screen genetic loci related to salt tolerance. Abel and MacKenzie^12^ employed eight bi-parental populations whose parents presented different chloride accumulation tolerances. The results indicated that the ratio of non-necrotic plants (tolerant) to necrotic plants (intolerant) was 3:1 in the F_2_ population, suggesting that chloride accumulation tolerance is controlled by a single dominant gene (*Ncl*). Using F_2:5_ lines derived from the cross of ‘S-100’ (tolerant) × ‘Tokyo’ (sensitive), Lee et al.^13^ discovered four stable QTLs located on Chromosome 3. The SSR marker Sat_091 might be associated with the *Ncl* salt tolerance allele, which accounts for more than 41% of the genetic variation in different environments.

Through F_2_ population crossed by cultivar Jackson and wild soybean accession JWS156-1, Hamwieh and Xu^14^ identified a dominant salt-tolerant QTL accounting for the salt tolerance rating; this QTL is located in the same region (Satt237–Satt255) as that reported by Lee et al.^13^, implying that the salt tolerance QTL is conserved in wild and cultivated soybeans. Hamwieh et al.^15^ used two RIL populations, and three sets of NILs confirmed a salt tolerance QTL on chromosome 3 reported by Lee et al.^13^ Hamwieh and Xu^14^ Ha et al.^16^ narrowed the salt tolerance QTL located on chromosome 3 reported in previous studies^12–15^, and the QTL was located in a 658 kb segment between SSR03_1335 and SSR03_1359. Using SCAR and Indel markers, Guan et al.^17^ mapped the salt tolerance QTL on chromosome 3 within 209 kb flanked by QS08064 and Barcsoyssr_3_1301.

In addition to a major salt tolerance QTL located on chromosome 3, Chen et al.^18^ used 184 lines from F_7:11_ RILs crossed by Kefeng No. 1 and Nannong1138-2 and detected a QTL between Sat_164 and Sat_358 on chromosome 18. In combination with 3.7 M SNP markers obtained from whole-genome resequencing and four salt tolerance-related traits (leaf scorch score, chlorophyll content ratio, leaf sodium content, and leaf chloride content), Do et al.^19^ detected a new minor salt tolerance locus on chromosome 8 via a panel of 234 accessions. In addition, Do et al.^20^ analyzed the leaf sodium content of 132 lines for F_2_ population derived from Williams 82 and Fiskeby III and detected a salt tolerance QTL on chromosome 13 with an LOD score of 4.6 and an R^2^ of 0.115. Zeng et al.^21^ employed F_4:6_ families derived from Osage and RA-452 and reported two other salt tolerance loci located on chromosome 13 and chromosome 15, which were identified in NaCl and KCl treatments, respectively. To identify QTLs governing soybean salt tolerance, Cho et al.^22^ used a mapping population derived from a cross between Cheongja 3 and IT162669 and a high-density genetic map containing 2,630 SNP markers. Two novel major salt tolerance loci, *qST6* and *qST10*, which are located on chromosome 6 and chromosome 10, respectively, were detected.

Although many salt-tolerant genetic loci have been reported, most of them have been obtained by linkage analysis via bi-parental populations. This is a major limitation for elite alleles mining because few recombination events occur in mapping populations. While multiple studies have reported quantitative trait loci (QTLs) linked to salt tolerance in soybean, these efforts face two key limitations: (1) the majority of QTLs correlate with only one or a limited trait of salt tolerance, (2) detection primarily relied on low-density linkage maps and traditional molecular markers. These shortcomings may compromise the precision of QTL position estimates and consequently diminish the utility of marker-assisted breeding. To this end, a natural population of 140 soybean accessions was evaluated for salt tolerance using the STIv index. On the basis of the 150 K SNP markers and “STIv”, the genetic components associated with soybean salt tolerance were dissected, which provided a theoretical basis for the breeding of salt-tolerant soybean plants via molecular breeding and pyramid breeding.

## Results

### Phenotypic variation and correlation of salt tolerance-related traits

To understand the phenotypic variation and correlation of the four salt tolerance-related traits, analysis of variance (ANOVA), descriptive statistics and correlation analysis were conducted for the soybean population. ANOVA revealed significant differences in four salt tolerance-related traits among the different accessions (Table 1). The maximum values of LA-STIv, PH-STIv, SFW-STIv and SDW-STIv were 2.02, 1.90, 0.71 and 1.14, respectively, which reached 20.20-, 6.13-, 11.83- and 9.50-fold greater than the corresponding minimum values. Further analysis revealed that the CVs of LA-STIv, PH-STIv, SFW-STIv and SDW-STIv were 67.94%, 36.60%, 49.71% and 59.60%, respectively. These results suggested that abundant genetic variations were existed in soybean natural population. Additionally, the absolute values of kurtosis and skewness for the salt tolerance-related traits indicated that all the traits presented normal distributions, implying that they were quantitative traits. In addition, correlation analysis revealed positive correlations among PH-STIv, SDW-STIv, and SFW-STIv, with correlation coefficients of 0.95 (SDW-STIv and SFW-STIv), 0.60 (SDW-STIv and PH-STIv) and 0.57 (PH-STIv and SFW-STIv), respectively (Figure S1).

**Table 1:** Phenotypic variation analysis of the salt resistance index in the natural population of soybean.

Trait	Mean	SD	Max	Min	CV (%)	Skew.^a^	Kurt.^b^	*P* value
LA-STIv	0.67	0.45	2.02	0.10	67.94	0.90	0.06	2.53 × 10^–26^
PH-STIv	0.90	0.33	1.99	0.31	36.60	0.26	−0.12	1.57 × 10^–14^
SFW-STIv	0.27	0.13	0.71	0.06	49.71	0.85	0.94	5.63 × 10^–12^
SDW-STIv	0.45	0.27	1.14	0.12	59.60	0.98	−0.03	3.27 × 10^–45^

^a^Skew. refer to skewness of natural population.

^b^Kurt. Refer to kurtosis of natural population.

### Genetic structure of soybean natural population

**Fig. 1:**
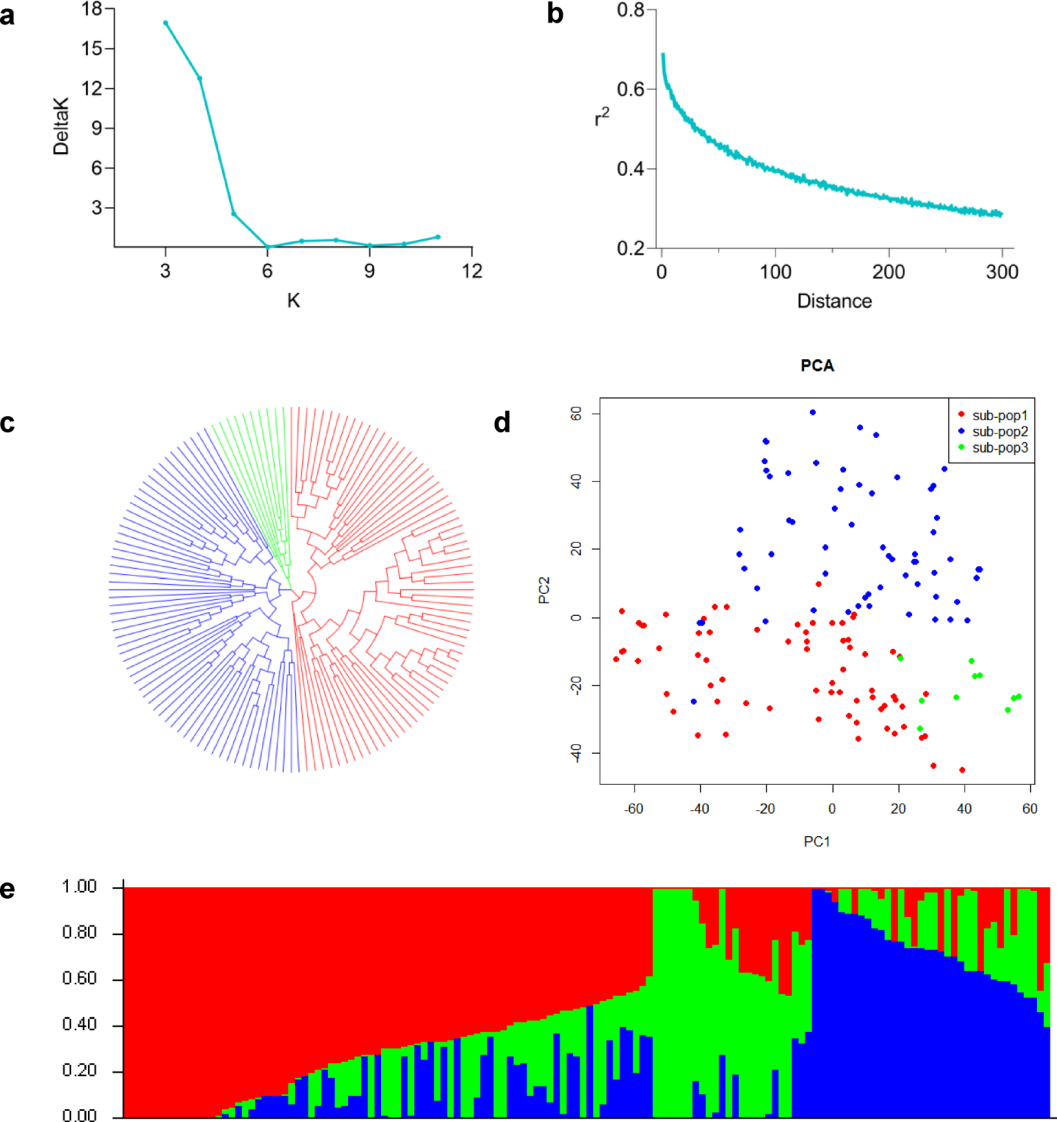
Population structure analysis of soybean. (**a**) delta K analysis. (**b**) LD decay analysis of the genome-wide average. (**c**) Phylogenetic tree analysis. (**d**) PCA plot of the first two components (PC1 and PC2). (**e**) Stack figure analysis of the population.

On the basis of the filtering criteria (missing ratio < 0.15 and minor allele frequency (MAF) ≥ 0.05), high-quality SNPs were selected. The population structure was subsequently analyzed on the basis of these SNPs. The results showed that ΔK reached its highest value when K = 3, indicating that the optimal number of subgroups was three (Fig. 1). The natural population was subsequently divided into three groups (sub-pop1, sub-pop2 and sub-pop3) (Fig. 1). Further analysis revealed that sub-pop1 included 69 germplasms, sub-pop2 included 61 germplasms, and sub-pop3 included 10 germplasms (Fig. 1). The PCA and phylogenetic tree of 140 accessions confirmed these results (Fig. 1). In addition, LD analysis demonstrated that the LD decreased to half of its maximum value at 150 kb for this population, which was a reference for candidate gene screening (Fig. 1).

### Identification of SNPs associated with salt tolerance in soybean

**Fig. 2:**
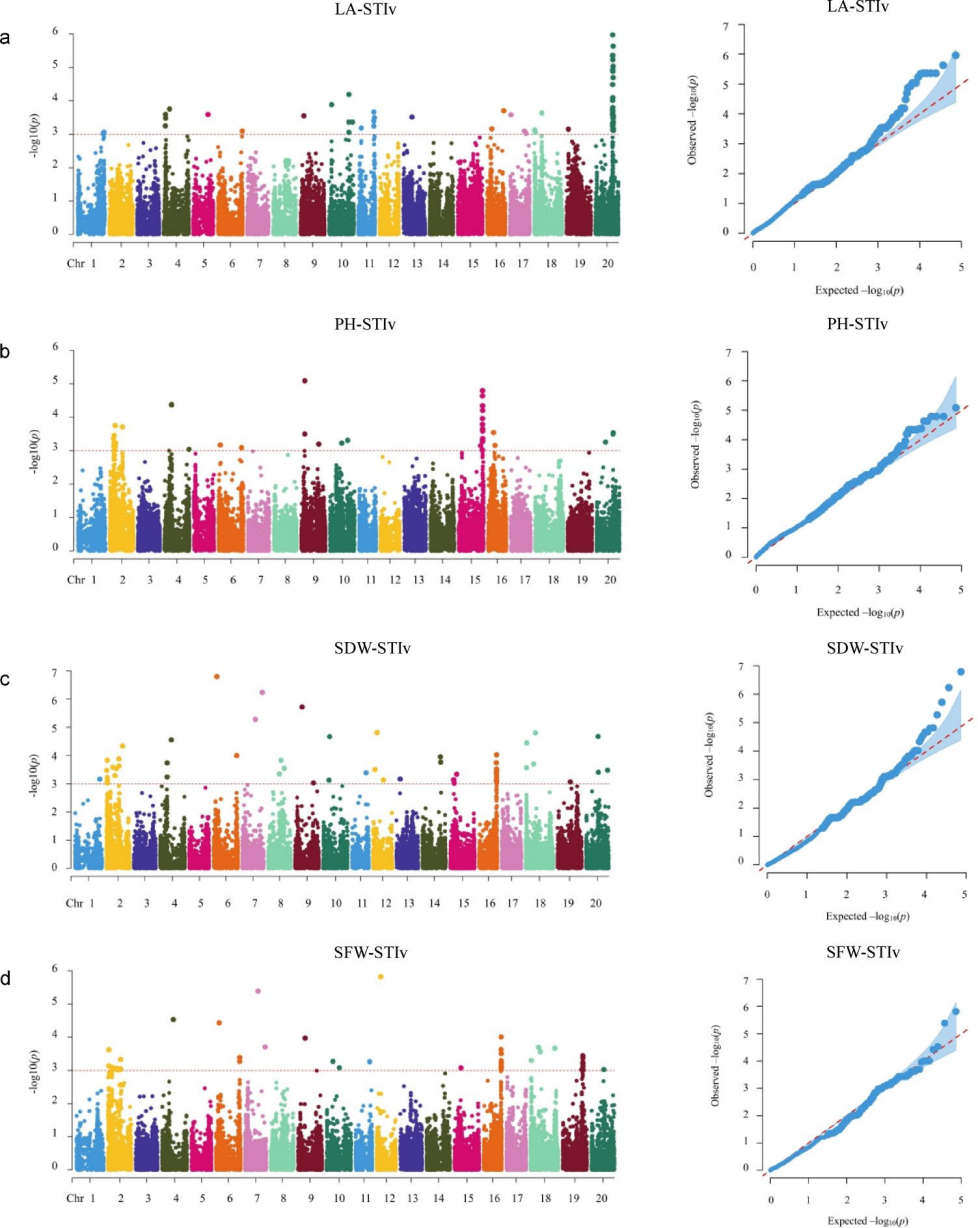
GWAS analysis of soybean salt tolerance in natural population. (**a**) Manhattan and QQ plots of LA-STIv. (**b**) Manhattan and QQ plots of PH-STIv. (**c**) Manhattan and QQ plots of SDW-STIv. (**d**) Manhattan and QQ plots of SFW-STIv.

To mine significant SNPs for salt tolerance, GWAS were performed based on four salt tolerance-related traits and 150 K SNPs. In total, 365 significant SNPs associated with soybean salt tolerance were detected, which were located on 19 chromosomes (except Gm03). Among them, 108 SNPs were associated with LA-STIv, 71 SNPs associated with PH-STIv, 95 SNPs associated with SDW-STIv and 91 SNPs associated with SFW-STIv (Fig. 2, Table S3). Further analysis revealed that 39 was located in the 5′UTR or 3′UTR and 43 was located upstream or downstream, which might regulate genes expressions. In addition, 141 were intergenic SNPs, 53 were intronic, 86 were exonic, and 3 were attributed to splicing sites (Fig. 2, Table S3). To further validate the accuracy of associated SNPs through regression analysis, 21 significant loci in the signal peaks were identified. These loci based on LD (150 kb) decay distance of the natural population, and were located on Gm02, Gm10, Gm11, Gm15, Gm16, Gm19, and Gm20 (Table 2).

**Table 2:** Significant loci associated with the signal peaks for soybean salt tolerance.

Locus	Trait	Chr	SNP interval	SNP number	Highest association SNP	−log_10_(*P*)
1	SFW-STIv;SDW-STIv	2	Gm02:1527027–Gm02:1648938	15	Gm02:1559363	3.03 –3.83
2	PH-STIv	2	Gm02:7622430–Gm02:7702874	5	Gm02:7622430	3.14
3	PH-STIv;SFW-STIv	2	Gm02:8501084;Gm02:8626991	8	Gm02:8600819	3.01–3.46
4	LA-STIv	10	Gm10:41250185–Gm10:41371943	9	Gm10:41250185	3.05–4.19
5	LA-STIv	11	Gm11:32366214–Gm11:32402605	7	Gm11:32384787	3.25–3.66
6	SDW-STIv	15	Gm15:6861171–Gm15:6997677	9	Gm15:6861171	3.14–3.15
7	PH-STIv	15	Gm15:49488731–Gm15:49633028	16	Gm15:49630767	3.30–4.79
8	PH-STIv	15	Gm15:49640970–Gm15:49785222	13	Gm15:49655283	3.27–4.79
9	PH-STIv	15	Gm15:49798519–Gm15:49844620	6	Gm15:49798519	3.29–4.21
10	SDW-STIv	16	Gm16:36012669–Gm16:36154637	14	Gm16:36100116	3.11–3.12
11	SDW-STIv;SFW-STIv	16	Gm16:36170007–Gm16:36287416	6	Gm16:36170007	3.02–3.11
12	SDW-STIv;SFW-STIv	16	Gm16:36586231–Gm16:36710889	5	Gm16:36682958	3.04–4.02
13	SFW-STIv	19	Gm19:41980035–Gm19:42127286	19	Gm19:42110952	3.05–3.48
14	SFW-STIv	19	Gm19:42132009–Gm19:42233967	11	Gm19:42233967	3.12–3.48
15	LA-STIv	20	Gm20:35654431–Gm20:35796837	8	Gm20:35779800	3.24–4.05
16	LA-STIv	20	Gm20:35970321–Gm20:36088187	7	Gm20:35975492	4.49–5.36
17	LA-STIv	20	Gm20:36321103–Gm20:36461144	7	Gm20:36366377	3.71–4.93
18	LA-STIv	20	Gm20:36570542–Gm20:36715934	8	Gm20:36695741	3.54–5.04
19	LA-STIv	20	Gm20:36724538–Gm20:36868573	8	Gm20:36823413	3.36–5.64
20	LA-STIv	20	Gm20:36877549–Gm20:37016269	9	Gm20:36940028	3.36–3.74
21	LA-STIv	20	Gm20:37036295–Gm20:37185025	6	Gm20:37046302	3.11–3.54

Furthermore, there were 69 SNPs on chromosome 20 divided into seven loci for soybean salt tolerance, of which 17 were located in exons (16 synonymous SNVs and one nonsynonymous SNV), seven in introns, seven in the 5′UTR or 3′UTR, and 11 in upstream or downstream. In addition, there were 65 SNPs on chromosome 2 divided into three loci. Among these 65 SNPs, 14 were located in exons, eight in introns, four in 5′UTRs or 3′UTRs, and 10 upstream or downstream. Furthermore, 52 SNPs were detected on chromosome 16, of which 19 were located in exons, 14 in introns, six in 5′UTRs or 3′UTRs, and three in upstream or downstream (Table S3).

Moreover, 50 SNPs on chromosome 15 were detected for soybean salt tolerance, which were attributed to four loci. Among these SNPs, seven were located in exons, eight in introns, four in 5′UTRs or 3′UTRs, and four in upstream or downstream (Table S3). Additionally, among the 365 SNPs, 48 SNPs show pleiotropic effects, which located on 12 chromosomes (Table S4). Among the 48 pleiotropic SNPs, 40 SNPs were associated with SFW-STIv and SDW-STIv, six SNPs were associated with SFW-STIv and PH-STIv, and two SNPs were associated with SDW-STIv and PH-STIv.

### Verification of significant SNPs for soybean salt tolerance

To verify the associated SNPs identified in this study, regression analysis was conducted between salt tolerance-related traits and the favorable allele numbers of 21 loci (Figure S2). The results revealed that the values of LA-STIv, PH-STIv, SDW-STIv and SFW-STIv were grown with increasing number of favorable alleles, which indicated that the more favorable alleles a soybean variety possessed, the greater its salt tolerance was. These results validated the reliability of the 21 salt tolerance loci identified in the present study.

Furthermore, to provide salt-tolerant soybean germplasms for breeding, 10 accessions with different salt tolerances were selected (the top five and bottom five in the natural population), and the results revealed that the five accessions with relatively high salt tolerance presented 13–15 favorable alleles at 21 loci, whereas the five accessions with lower salt tolerance presented 1–3 favorable alleles (Table S5). Moreover, except for Henong No. 63, the other tolerant accessions belonged to sub-pop1, whereas the sensitive accessions (except Heihe No. 36) were attributed to sub-pop2. Further analysis found that no germplasm contained 21 favorable alleles, which indicated that many genetic improvements are needed in the breeding of salt tolerance in soybean (Table S5). These results not only confirmed the reliability of the significant SNPs detected in the present study but also provided elite germplasms for use in salt tolerance breeding programs.

### Screening causal genes responsible for salt tolerance in soybean

To identify potential salt-tolerant candidate genes, we employed 365 associated SNPs to detect causal genes. In total, 333 genes were identified in the flanking region (150 kb) of sigificantly associated SNPs, of which 72 genes showed SNP mutations in exons, 35 genes owned the 5′UTR or 3′UTR SNP mutations, 37 genes possessed upstream or downstream SNP mutations, 202 genes have intergenic SNP mutations, and 40 genes held intronic SNP mutations (Table S6). To filter salt tolerance causal genes, RNA expression and gene functional annotations were employed. Based on differentially expressed genes (DEGs) reported in publicly available dataset GSE237798 from NCBI (https://www.ncbi.nlm.nih.gov/geo/query/acc.cgi?acc=GSE237798), 113 genes were selected (Table S6). Subsequently, according to Wm82 gene annotations in SoyBase (https://www.soybase.org/), nine genes related to soybean salt tolerance that participate in the salt stress response, xylem development, the regulation of the ethylene biosynthetic process, the flavonoid biosynthetic process and photosynthetic electron transport were identified (Table 3, Figure S3). Among them, *Glyma.04G044900* (based on Gm04:3588887), *Glyma.10G058300* (based on Gm10:5337835), *Glyma.11G242200* (based on Gm11:33630597), *Glyma.20G128100* (based on Gm20:36973722 in locus20) and *Glyma.20G129600* (based on Gm20:37078097 in locus 21) were detected from LA-STIv; *Glyma.19G159400* (based on Gm19:42025090 and Gm19:42016158 in locus 13) and *Glyma.19G161100* (based on Gm19:42180039 and Gm19:42189574 in locus13) were detected from SFW-STIv; *Glyma.15G263700* (based on Gm15:49708002, Gm15:49710598, Gm15:49714298, Gm15:49727153, Gm15:49729603, Gm15:49733535 and Gm15:49738395 in locus8) was detected from PH-STIv; and *Glyma.16G199100* (based on Gm16:36046290 and Gm16:36050536 in locus10)was detected from SDW-STIv (Table S6).

**Table 3:** Causal genes associated with salt tolerance in soybean.

Causal gene	Annotation	Biological process description
*Glyma.04G044900*	C2H2 zinc-finger protein	response to salt stress
*Glyma.10G058300*	Ethylene signal transduction protein	response to salt stress
*Glyma.11G242200*	Homeobox-leucine zipper protein	response to salt stress
*Glyma.15G263700*	MYB family transcription factor APL-like	xylem development
*Glyma.16G199100*	Per1-like family protein	response to salt stress
*Glyma.19G159400*	Ethylene overproducer-like protein	regulation of ethylene biosynthetic process
*Glyma.19G161100*	Auxin-induced protein	response to auxin stimulus
*Glyma.20G128100*	Limonoid UDP-glucosyltransferase	flavonoid biosynthetic process
*Glyma.20G129600*	STRESS ENHANCED protein	photosynthetic electron transport in photosystem I

### Analysis of favorable genotyping for causal genes

**Fig. 3:**
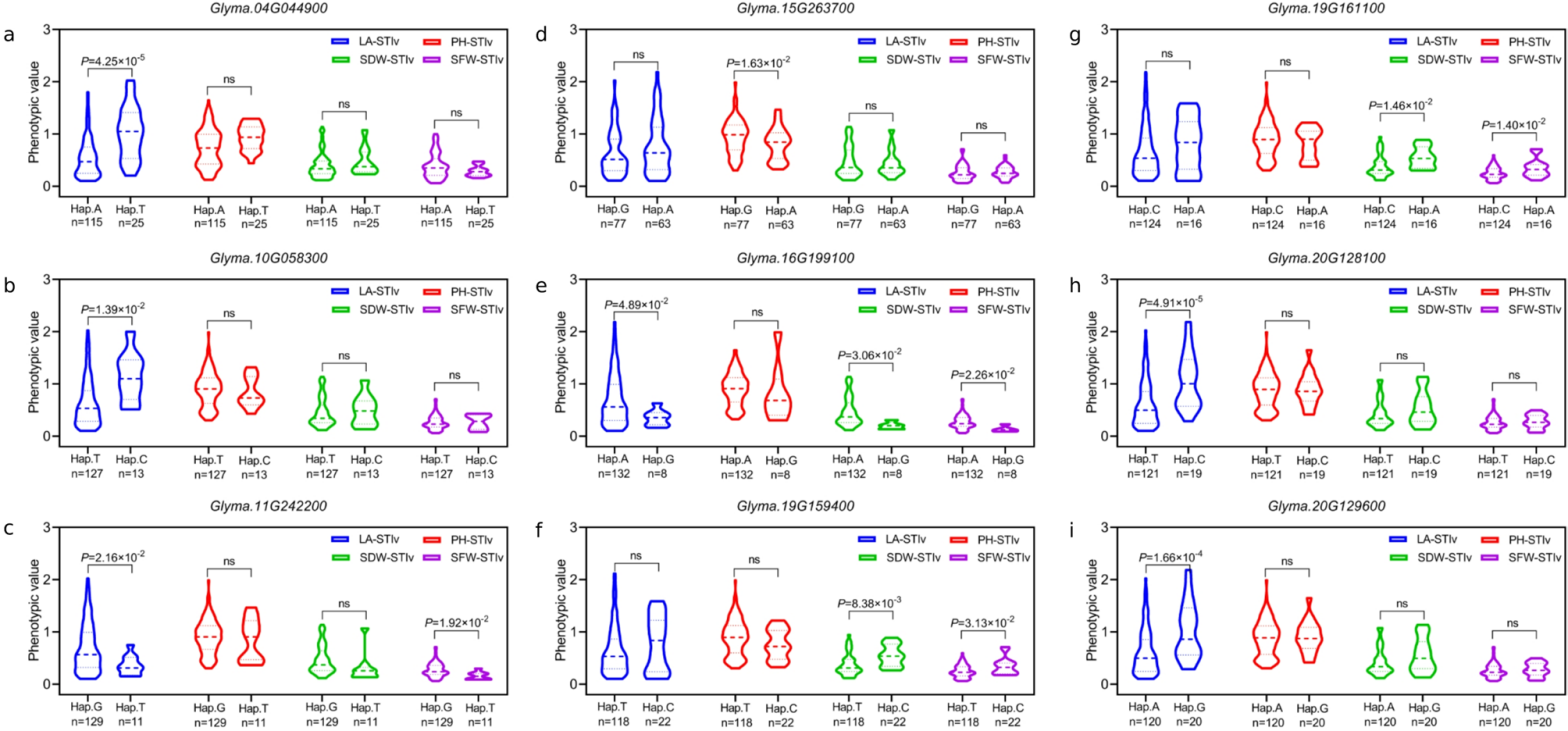
Haplotype analysis of candidate genes. (**a**) *Glyma.04G044900*. (**b**) *Glyma.10G058300*. (**c**) *Glyma.11G242200*. (**d**) *Glyma.15G263700*. (**e**) *Glyma.16G199100*. (**f**) *Glyma.19G159400*. (**g**) *Glyma.19G161100*. (**h**) *Glyma.20G128100*. (**i**) *Glyma.20G129600*. Green violin: LA-STIv; blue violin: SH-STIv; red violin: SDW-STIv; purple violin: SFW-STIv. Statistical significance was assessed via a two-tailed *t*-test.

**Fig. 4:**
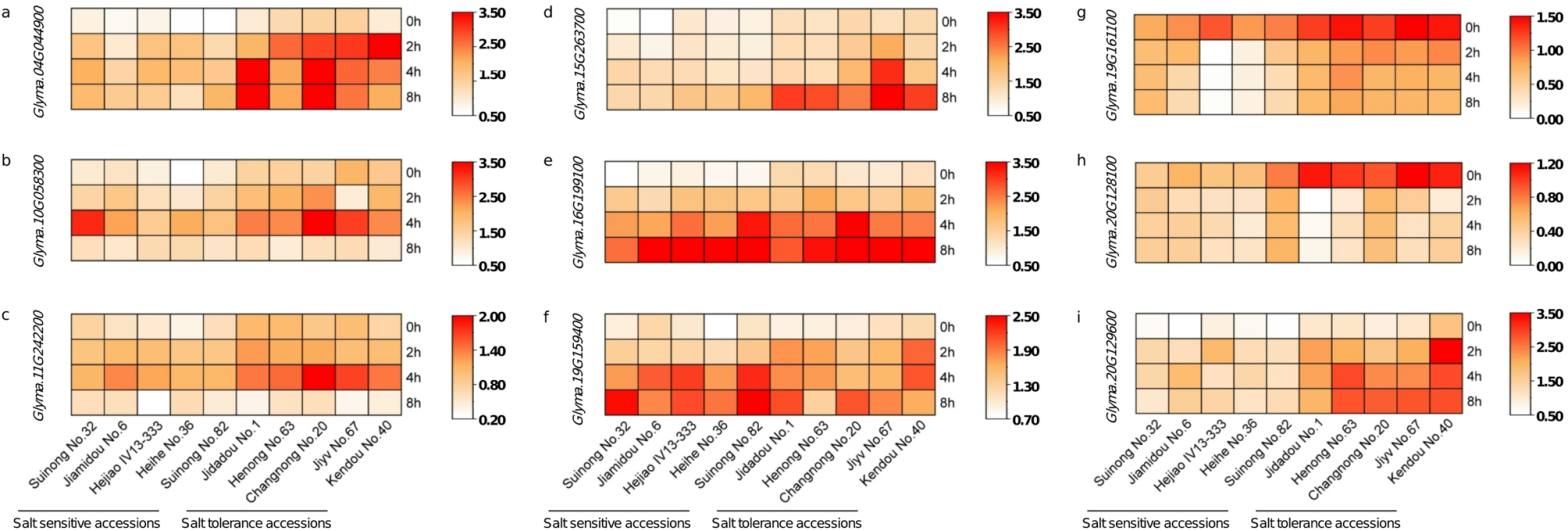
Expression pattern analysis of candidate genes. (**a**) Expressions levels of *Glyma.04G044900* in different accessions at 0, 2, 4 and 8 h after salt stress. (**b**) Expressions levels of *Glyma.10G058300* in different accessions at 0, 2, 4 and 8 h after salt stress. (**c**) Expressions levels of *Glyma.11G242200* in different accessions at 0, 2, 4 and 8 h after salt stress. (**d**) Expressions levels of *Glyma.15G263700* in different accessions at 0, 2, 4 and 8 h after salt stress. (**e**) Expressions levels of *Glyma.16G199100* in different accessions at 0, 2, 4 and 8 h after salt stress. (**f**) Expressions levels of *Glyma.19G159400* in different accessions at 0, 2, 4 and 8 h after salt stress. (**g**) Expressions levels of *Glyma.19G161100* in different accessions at 0, 2, 4 and 8 h after salt stress. (**h**) Expressions levels of *Glyma.20G128100* in diffrent accessions at 0, 2, 4 and 8 h after salt stress. (**i**) Expressions levels of *Glyma.20G129600* in different accessions at 0, 2, 4 and 8 h after salt stress.

Gene annotation analysis revealed that the causal gene *Glyma.04G044900* encodes a C_2_H_2_ zinc-finger protein. Meanwhile, this gene possesses one SNP in an exon, which divided the natural population into two haplotypes (Hap. A, Hap. T), and a significant difference in LA-STIv was detected between the haplotypes (Fig. 3, Table S6). The analysis of expression suggested that *Glyma.04G044900* exhibited a progressive increase under salt stress, showing significant different between salt-sensitive and tolerance accessions (Fig. 4). For the candidate gene *Glyma.10G058300* ecoding ethylene signal transduction protein, significant differences in LA-STIv were also detected between the haplotypes divided by intergenic SNPs in *Glyma.10G058300* (Fig. 3). The expression pattern of *Glyma.10G058300* exhibited an initial increase followed by subsequent decline. The candidate gene *Glyma.11G242200*, encoding a homeobox-leucine zipper protein, is also responsible for soybean salt tolerance. Two haplotypes divided by SNPs in the 3′UTR were significantly different in LA-STIv and SWF-STIv (Fig. 3). The expression of *Glyma.11G242200* reached highest point at the 4 h under salt stress (Fig. 4).

The causal gene *Glyma.20G128100* on chromosome 20 encoded the limonoid UDP-glucosyltransferase and participated in the flavonoid biosynthetic process, which might be responsible for soybean salt tolerance. There was one SNP in the 5′UTR of *Glyma.20G128100*, which divided the natural population into two haplotypes (Hap. T, Hap. G), with a significant difference in LA-STIv. The expression pattern of *Glyma.20G128100* decreased significantly after 2 h of salt stress (Fig. 4). Moreover, gene annotation analysis suggested that *Glyma.20G129600* encoded a short ENHANCED protein and was involved in photosynthetic electron transport in photosystem I, which might be participated in salt stress. In terms of SNPs in the 3′UTR, two haplotypes of *Glyma.20G129600* were detected, with significant differences in LA-STIv (Fig. 3). The expression patterns analysis showed that salt stress triggered rapid upregulation of *Glyma.20G129600*, showing significant differences across salt-tolerant and salt-sensitive accessions (Fig. 4).

In addition, two causal genes were identified on the basis of SFW-STIv. *Glyma.19G159400* encoded an ethylene overproducer-like protein, and regulated the ethylene biosynthetic process. The SNP in the 5′UTR of *Glyma.19G159400* divided natural population into two haplotypes (Hap. T, Hap. C), showing significant difference in both SDW-STIv and SFW-STIv, validating the correlation results. The gene expression levels of *Glyma.19G159400* were induced by salt stress in ten soybean accessions with different salt tolerance (Fig. 4). *Glyma.19G161100* has one intergenic SNP and one intronic SNP, which was predicted to encode an auxin-induced protein and respond to auxin stimulus. The intronic SNP divided population into two haplotypes (Hap. C, Hap. A), also showing significant difference in SDW-STIv and SFW-STIv, consistent with the correlation analysis. (Fig. 3). Furthermore, a significant downregulation of *Glyma.19G161100* expression was observed following 2-h salt stress exposure (Fig. 4).

According to PH-STIv, *Glyma.15G263700* was found, which encoded MYB family transcription factor and involved in xylem development. There were six intergenic SNPs and one intronic SNP in *Glyma.15G263700*. Significant differences in PH-STIv were also detected between the haplotypes according to any of the seven SNPs in *Glyma.15G263700.* The expression levels of *Glyma.15G263700* were significant increased in salt tolerance accessions (Fig. 4). *Glyma.16G199100* was another salt tolerance gene associated with SDW-STIv that was predicted to encode a Per1-like family protein and participate in soybean salt tolerance. Two SNPs were identified in *Glyma.16G199100*, which divided the natural population into two haplotypes, with significant differences in SDW-STIv, SDW-STIv and LA-STIv (Fig. 3). *Glyma.16G199100* expression levels showed significant upregulation after 2, 4, and 8 h of salt stress.

## Discussion

### Mining tolerant varieties for salt stress in natural population

Salt stress is a major abiotic stress that hampers soybean physiology and even threatens growth and production^23–25^. In turn, soybean has evolved acclimatory or adaptive responses to salt stress^26^. Elite germplasms constitute the basis for analyzing the salt tolerance adaptability of soybean, which is an effective way to dissect the resistance of soybean plants to salt stress. The variety salt tolerance index (STIv) not only taken into account the salt sensitivity of varieties but also focus on the rank of varieties for different traits in natural population under salt stress, which is suitable for evaluating soybean salt tolerance. Furthermore, under salt stress, the height, leaf area, and biomass of soybean plants significantly decreased. Thus, the STIv values of plant height, leaf area, shoot fresh weight and shoot dry weight were used to evaluate soybean salt tolerance. According to the STIv values of different traits, five tolerant accessions with relatively high STIv values, namely, Henong No. 63, Jidadou No. 1, Changnong No. 20, Jiyv No. 67 and Kendou No. 40, were screened, four of which were sub-pop1. Further analysis found that five tolerant accessions presented 13–15 favorable alleles at 21 loci, whereas five sensitive accessions presented 1–3 favorable alleles. These elite germplasms not only provided the basis for molecular mechanism dissection of soybean salt tolerance but also offered a theoretical basis for parent selection in soybean breeding. To validate the accuracy of salt-tolerance identification, the comprehensive salt tolerance coefficient (*D*-value) was employed to evalute the salt tolerance of natural population. Based on *D*-value, Dongnong 63 was classified as a salt-sensitive accession, which was consistent with previous study, confirming the reliability of our methodolog^27^.

### Significant loci associated with soybean salt tolerance

At present, major genetic loci for soybean salt tolerance have been detected on chromosome 3 via bi-parental populations which are known for their few recombination events in mapping populations, and most of them focus on ordered traits, such as salt tolerance rating, leaf scorch and plant survival^13–18^. To discover novel genetic loci associated with soybean salt tolerance, the STIv of four traits and 150 K SNPs were employed to conduct GWAS analysis. On the basis of the significant signal peaks, 21 loci located on chromosomes 2, 10, 11, 15, 16, 19, and 20 were identified. Compared with previous studies, Locus 14 detected from LA-STIv overlapped with *Leaflet area 1-g1.2*, *Leaflet length 1-g1.2*, and *Leaflet width 1-g2.2*^28^. Locus 17 overlapped with *Leaflet width 1-g2.3*^28^. Locus 7 identified from PH-STIv overlapped with *Plant height 3-g16*^29^. Additionally, the significant SNP Gm02:1,734,308 identified in this study was located 45 kb from *Salt tolerance 1-g13*^1^. The other significant SNP Gm15:7068549 was in *Salt tolerance 8-2* and *Salt tolerance 8-4*^21^.

### Causal genes for soybean salt tolerance

To uncover novel functional genes responsible for soybean salt tolerance, the candidate genes flanking significant regions were analyzed based on gene annotations, DNA mutations, and RNA expression patterns. The results revealed nine causal genes related to salt tolerance. Among these genes, *Glyma.04G044900,* encoding a C_2_H_2_ zinc-finger protein (ZFP), was predicted to participate in the soybean salt stress response. C_2_H_2_ ZFP plays vital roles in the salt stress tolerance of many plants, such as cucumber, apple, rice and wheat^30–33^. The overexpression of *MdZAT17* reduced the MDA content and ROS accumulation and increased the anthocyanin concentration under salt stress, which indicated that *MdZAT17* plays a positive role in salt tolerance^31^. *OsZFP179* overexpression transgenic plants exhibited the accumulation of proline, soluble sugars, SOD and POD, leading to increased salt tolerance^34,35^. Compared with the RNAi plants, the transgenic rice of *OsZFP213* presented increased salt tolerance, which resulted in an increased ability to scavenge reactive oxygen, such as SOD, APX, and CAT^36^. In addition, ectopic expression of *TaZNF* significantly improved salt tolerance in *Arabidopsis*^37^. Expression pattern analysis revealed that *Glyma.04G044900* displayed salt-stress-inducible upregulation, with significantly higher transcript levels in salt-tolerant accessions compared to sensitive accessions. Thus, we deduced that the causal gene *Glyma.04G044900* might be involved in soybean salt tolerance.

Under salinity stress, various secondary metabolites produced by plants accumulate to adapt to the environment^38,39^. The FLS (flavonol synthase) gene family encodes key enzymes in the flavonoid biosynthesis pathway that catalyze flavonol production, including quercetin, kaempferol, and myricetin^40^. Flavonoids are important secondary metabolites that are known for their excellent ability to scavenge reactive oxygen species (ROS) during abiotic stress responses^41^. Compared with wild-type, *MsFLS13*-overexpressing transgenic alfalfa presented greater total flavonoid content and higher antioxidant capacity under saline conditions, which indicated that *MsFLS13* enhanced the tolerance of alfalfa to salt stress^42^. Under salt stress, the overexpression of *AvFLS* led to a significant increase in flavonol content and antioxidant enzyme activity in tobacco^43^. The overexpression of *EkFLS* in *Arabidopsis* improved flavonoid accumulation, POD contents and SOD contents to protect the plants. In contrast, silencing *GhFLS1* (flavonol synthase gene) in cotton present a decrease in proline content and salt sensitivity^44^, and the silencing of *BrFLS1* or *BrFLS3.3* reduces the antioxidant capacity and salt tolerance in Brassica vegetables^45^. In this study, the causal gene *Glyma.20G128100*, which participated in the flavonoid biosynthetic process, was detected and might produce flavonoids to against salt tolerance. Haplotype analysis revealed that the Gm20:36973722 located in the 5′UTR of *Glyma.20G128100* divided the population into two haplotypes, suggesting that this variation may regulate *Glyma.20G128100* expression. Additionally, the transcript level of *Glyma.20G128100* was markedly reduced following 2 h of salt stress, and significant differences were observed among sensitive and tolerance accessions.

The mutant of the ethylene synthesis gene *ACS7* presented increased salt tolerance, and the transcript levels of the stress-responsive genes involved in the ABA pathway were increased in *acs7*, which suggested that acs7 might participate in the salt response via the ABA-dependent pathway. In addition, the causal gene *Glyma.19G159400* was predicted to regulate the ethylene biosynthetic process, which might act as a negative regulator in salt tolerance^46^. The transcriptional activity of *Glyma.19G159400* was enhanced by salt stress in ten soybean genotypes exhibiting differential salt tolerance. Moreover, *Glyma.10G058300*, which encodes an ethylene signal transduction protein, was detected on chromosome 10 in this study. The homologous gene of *Glyma.10G058300* in *Arabidopsis* (*AT5G03280*, EIN2) encoded a central membrane protein involved in ethylene signaling. Mutation of EIN2 results in extreme salt sensitivity, suggesting that EIN2 is required for salt tolerance^47^. The expression level of *Glyma.10G058300* involved an initial rise and a subsequent reduction in present study.

Furthermore, the candidate gene *Glyma.11G242200* encoded a homeobox-leucine zipper protein, which are crucial for plant growth, development, and response to abiotic stresses^48^. Previous study demonstrated that homeobox-leucine zipper protein improved salt tolerance in *Arabidopsis* and oilseed rape^49,50^. The expression level of *Glyma.11G242200* peaked at 4 h under salt stress conditions, showing signifcant difference between salt-sensitive and salt-tolerance accessions. The efficiencies of photosystem I (PI) and photosystem II (PII) are usually inhibited by salt stress^51–54^. And photosynthesis in chloroplasts was disrupted by salt stress^55,56^. The candidate gene *Glyma.20G129600* was predicted to participate in photosynthetic electron transport, which might be involved in the salt stress response. Expression profiling revealed that *Glyma.20G129600* was rapidly upregulated in response to salt stress, with distinct expression patterns observed between salt-tolerant and salt-sensitive genotypes.

Among the nine candidate genes, four were associated with leaf area. As a organ of plants, leaves are easily accessible for sampling and measuring morphological indicators. They are highly responsive to salt stress and can rapidly reflect the salt tolerance capacity of plant. Under salt stress, the plant leaves turned yellow, chloroplast was degraded and photosynthesis was inhibited. Shelke et al. demonstrated that under different salt (NaCl) treatments, leaf area act as key determinants of salt tolerance in soybean, regulating net photosynthesis and carbon allocation to maintain plant growth under stress^57^. In this study, candidate gene *Glyma.20G129600* encoded a short ENHANCED protein and involved in photosynthetic electron transport in photosystem I. On the other hand, salt stress induces a burst of ROS, triggering massive production of secondary metabolites such as flavonoids in leaves, which scavenge ROS. The causal gene *Glyma.20G128100* participated in the flavonoid biosynthetic process might be involved in salt tolerance through ROS scavenging.

In summary, a high-throughput, low-cost targeted SNP genotyping chip could be developed based on the 365 significantly associated SNPs detected in this study. This platform enables precise salt-tolerance evaluation in soybean germplasm through marker-assisted selection, thereby accelerating the development of salt-tolerant cultivars. The findings in this paper provided both theoretical foundations and material resources for soybean salt-tolerance breeding. To advance this work, three key future directions are proposed: (1) The salt-tolerance phenotyping should be systematic evaluated in saline-alkali pool across critical growth phases. (2) CRISPR/Cas9-mediated editing could be employed to characterize candidate gene functions. (3) The salt-tolerance loci detected in this study and other high yield or quality loci should be integrated in future soybean pyramiding breeding.

## Materials and methods

### Plant materials and salt stress design

A core set of 140 soybean accessions was selected from *National Central Asian Characteristic Crop Germplasm Resources Medium-term Gene Bank (Urumqi)*, which were listed in Table S1.

The study was conducted at Xinjiang Zepu Soybean Breeder Base (N 39°58′, E 77°04′, China). The nutritional soil (peat:perlite:leaf mold at a ratio of 5:3:2, v:v:v) and field soil were evenly mixed at a 1:1 ratio. The mixed soil was subsequently placed in a square plastic pot (18 cm × 18 cm), with 2 L of water added. Nine seeds of each accession were planted in square plastic pot (18 cm × 18 cm) with 2–4 cm soil cover. Seven days after sowing, the plants were thinned, and five uniform plants for each accession were retained. Two treatments were subsequently conducted at the V1 stage (Vegetative 1, refers to the soybean growth stage characterized by complete expansion of the first trifoliolate leaf), with the CK (control group) supplemented with water and the salt treatment supplemented with 1.50% NaCl solution^58–60^. A randomized complete block design was used for each treatment. Three replications were performed.

### Evaluation of salt tolerance in the soybean population

Twenty days after treatment, three plants in each pot were selected to assess soybean salt tolerance. In accordance with the “*Specification and Data Standard of Soybean Germplasm Resources Description*”, four salt tolerance-related traits, such as plant height (PH), leaf area (LA), shoot fresh weight (SFW) and shoot dry weight (SDW), were measured for each treatment. According to the formula STIv = (P_w_/P_mw_)*(P_s_/P_ms_)*(P_ms_/P_mw_), soybean salt tolerance was evaluated, where P_w_ is the phenotype value under CK conditions, P_s_ is the phenotype value under salt treatment, P_mw_ is the mean value of the natural population under CK conditions, and P_ms_ is the mean value of the natural population under salt treatment^61^. Based on membership function analysis, the comprehensive salt tolerance coefficient (*D*-value) of soybean seedlings was calculated, and their salt tolerance grade was determined by the *D*-value^62^. The salt tolerance was evluated by salt tolerance grade which was classified based on the *D* value, with the criteria as follows: Salt intolerance (0.00–0.20), Salt-sensitive (0.21–0.40), Moderate salt tolerance (0.41–0.60), Salt tolerance (0.61–0.80), and Strong salt tolerance (0.81–1.00).

### Statistical analysis of phenotypes

The analysis of variance (ANOVA), phenotypic variation and correlation coefficient of the four salt tolerance-related traits were analyzed via SPSS version 25.0 software.

### Genotype and association analysis

The natural population was genotyped with SoySNP150K (Zhongdouxin-1), which was analyzed by Beijing Compass Biotechnology Company Limited. The SNPs of “Zhongdouxin-1” were selected from re-sequencing data of 2533 soybean accessions. The high-quality reads were mapped to Williams82 (Wm82) reference genome (GlymaWm82.a2.v1) via BWA software. And SNPs variant were identified through GATK-3.8. SNPs annotation were performed using ANNOVAR software based on the Wm82.a2.v1 soybean reference genome. This work was completed through a collaboration between the Chinese Academy of Agricultural Sciences and Compass Biotechnology Company Limited.

According to a missing ratio < 0.15 and minor allele frequency (MAF) ≥ 0.05, the SNPs were filtered. On the basis of these high-quality SNPs, population structure, kinship, and GWAS were subsequently conducted. The population structure was analyzed via STRUCTURE 2.3.4 software, with the K value (number of subgroups) set from 1 to 11, a burn-in of 10,000 and a run length of 100,000, and each K with five independent runs^63^. The Q matrix was subsequently obtained under the highest ΔK value. Additionally, on the basis of polymorphic SNPs, PCA and kinship analysis were performed via TASSEL 5.0 software, and a phylogenetic tree was constructed via this software^64^.

Moreover, linkage disequilibrium (LD) decay was estimated via Plink 1.9 software. The LD decay distances were selected at half of the maximum value for r^2^. To dissect the genetic component of soybean salt tolerance, a GWAS was conducted via TASSEL 5.0 software with a mixed linear model (MLM). The *P* value was set as 0.001. According to a previous study, associated SNPs with physical distances of less than 150 kb (LD decay distance) were considered to be the same locus^65^. If the physical distance between SNPs was smaller than LD distance (150 kb), the SNPs were assigned to the same locus. A filtering threshold was applied during locus selection, whereby locus containing fewer than five SNPs weas discarded.

### Validation of salt tolerance associated SNPs

To verify the SNPs associated with salt tolerance, the soybean accessions were classified on the basis of the number of favorable SNPs with the greatest significance at each locus. Regression analysis between favorable allele numbers and soybean salt tolerance was conducted via SPSS V25.0 software^66^.

### Screening of causal genes for soybean salt tolerance

To screen out causal genes for soybean salt tolerance, candidate genes were first selected from the flanking regions of significantly associated SNPs. Candidate genes were subsequently filtered on the basis of gene function annotation in SoyBase (https://www.soybase.org/). The gene expression levels were subsequently analyzed on the basis of published soybean salt tolerance transcriptome data from Gene Expression Omnibus (GEO), under the following accession number: GSE237798 (https://www.ncbi.nlm.nih.gov/geo/query/acc.cgi?acc=GSE237798)^67^. On the basis of these findings, causal genes related to soybean salt tolerance were screened out.

To detect expression patterns of the nine candidate genes, ten soybean accessions were selected from a natural population, comprising five salt sensitive accessions (Suinong No. 32, Jiamidou No. 6, Hejiao IV13-333, Heihe No. 36, Suinong No. 82) and five salt tolerance accessions (Jidadou No. 1, Henong No. 63, Changnong No. 20, Jiyv No. 67, Kendou No. 40). Thirty seeds from each accession were germinated using the same method described above. Leaf samples were collected at 0, 2, 4, and 8 h after salt stress, with three samples per time point.

Total RNA from different soybean accessions was extracted using a plant RNA Extraction Kit (Tiangen, China), and the first-strand cDNA was performed using Primer Script RT Reagent Kit (Takara, Japan) following to the manufacturer’s instruction. LightCycler 480 Real-Time System (Roche, Switzerland) was employed to analyse the expression levels for nine candidate genes with the *Actin* (*Glyma.18G290800*) gene as an internal control. The expressions of candidate genes were standardized using *GmActin* and calculated as 2^−ΔΔCt^ method^68^. The qRT-PCR primers were listed in Table S2.

## Conclusion

In total, 365 SNPs on 19 chromosomes (excluding Gm03) were found to be associated with soybean salt tolerance, of which 108 SNPs were associated with LA-STIv, 71 SNPs were associated with PH-STIv, 95 SNPs were associated with SDW-STIv, and 91 SNPs were associated with SFW-STIv. In addition, there were 69 SNPs divided in seven loci on chromosome 20, 65 SNPs in three loci on chromosome 2, and 52 SNPs in two loci on chromosome 16. Furthermore, on the basis of gene functional annotations, SNP mutations, and RNA expression, nine causal genes responsible for soybean salt tolerance, which participated in the salt stress response, xylem development, the regulation of the ethylene biosynthetic process, the flavonoid biosynthetic process and photosynthetic electron transport, were identified.

## Electronic supplementary material

Below is the link to the electronic supplementary material.

Supplementary Material 1

Supplementary Material 2

## Data Availability

All data generated or analyzed during this study are included in this article and its supplementary information files.
