# Supplementary material for: Mining genetic loci and candidate genes related to salt tolerance traits in soybean
Source: Sci Rep. 2025 Jul 23;15:26826. doi: 10.1038/s41598-025-08702-y (PMC12287287; doi:10.1038/s41598-025-08702-y)
Supplement: Supplementary file 1 — Supplementary Material 1 [file 41598_2025_8702_MOESM1_ESM.docx]

**Figure S1
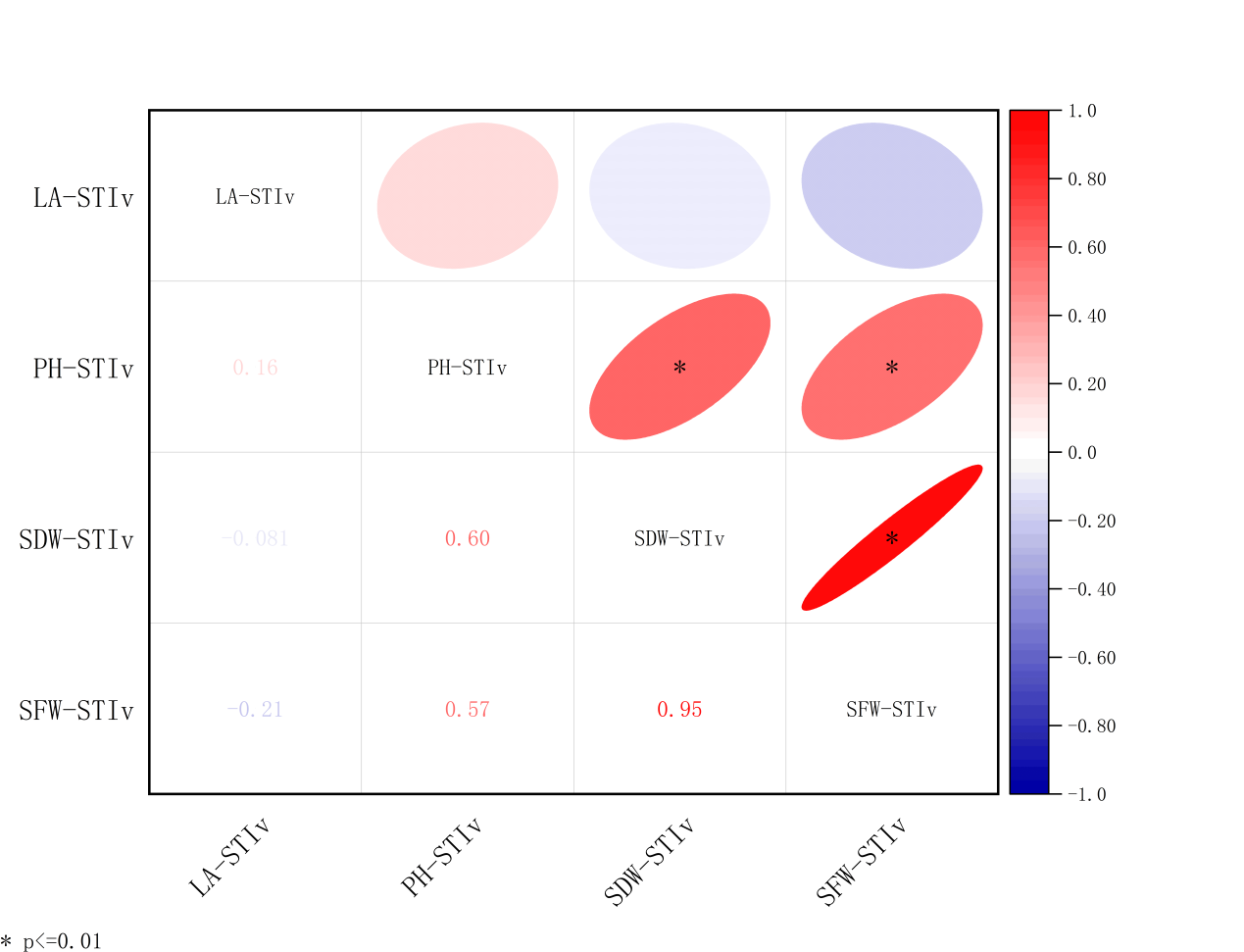
**

**Fig. S1** Correlation analysis of salt tolerance-related traits, *P* < 0.01.

**
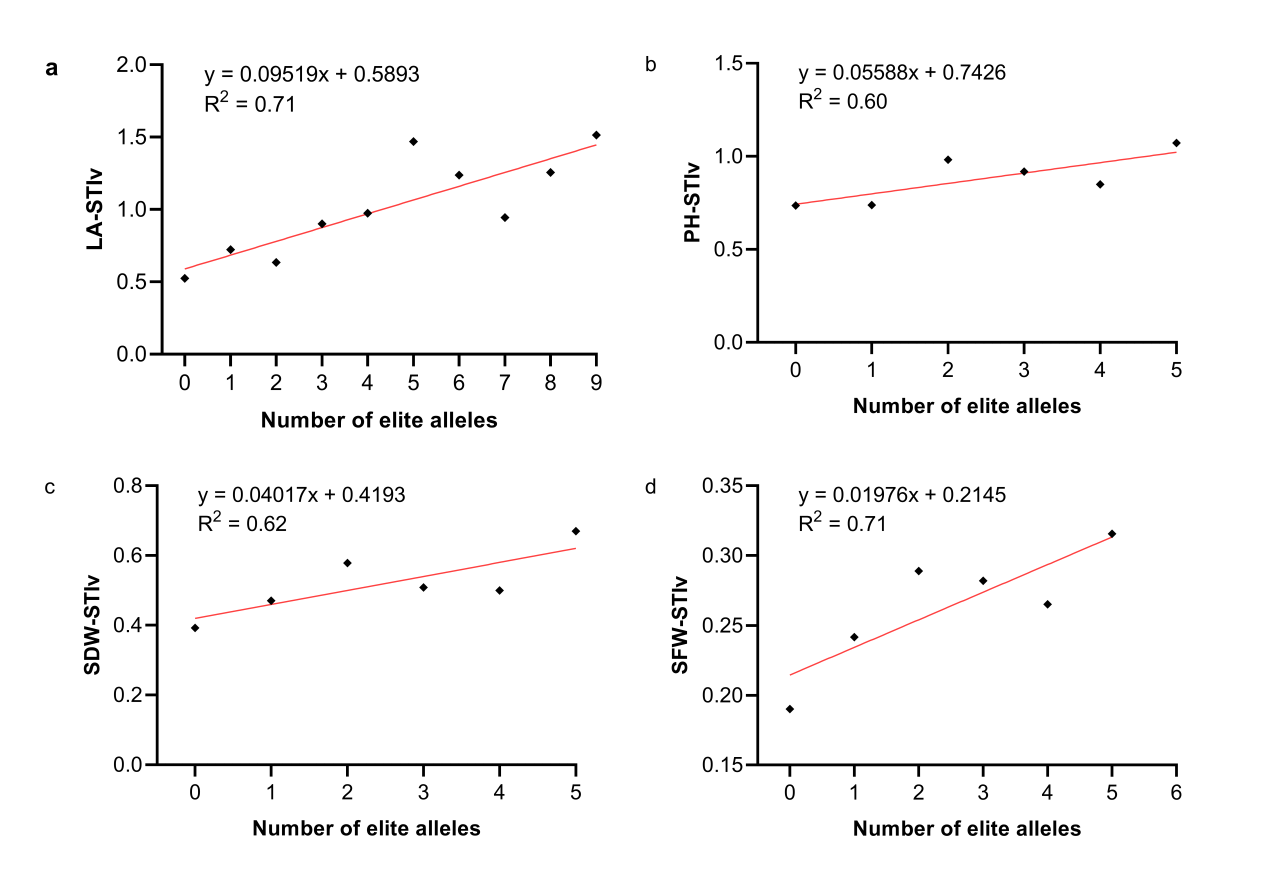
Figure S2**

**Fig. S2** Regression analysis of salt tolerance-related traits and elite allele numbers in soybean accessions. X-axis indicates the numbers of elite alleles; Y-axis is the phenotype value of soybean accessions.

**
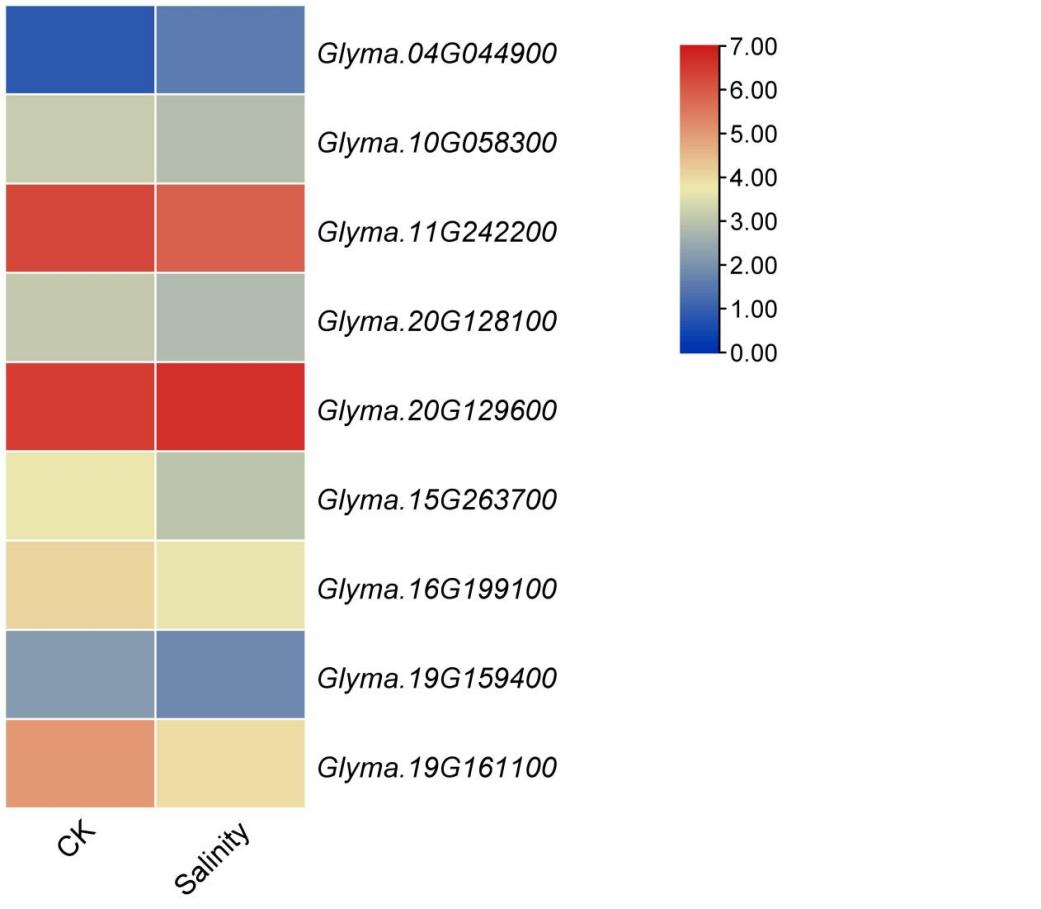
Figure S3**

**Fig. S3** Expressions of candidate genes involved in soybean salt tolerance.

Data from the published soybean salt stress transcriptome sequencing in NCBI (GSE237798).
